# The MAPK pathway as an apoptosis enhancer in melanoma

**DOI:** 10.18632/oncotarget.2079

**Published:** 2014-06-08

**Authors:** Johannes M. Haydn, Anita Hufnagel, Johannes Grimm, Katja Maurus, Manfred Schartl, Svenja Meierjohann

**Affiliations:** ^1^ Department of Physiological Chemistry, Biocenter, University of Wurzburg, Wurzburg, Germany; ^2^ Comprehensive Cancer Center Mainfranken, University Hospital Wurzburg, Germany

**Keywords:** Crosstalk, chemotherapy resistance, RAS, PI3K, melanoma

## Abstract

Inhibition of RAF/MEK/ERK signaling is beneficial for many patients with BRAF^V600E^–mutated melanoma. However, primary and secondary resistances restrict long-lasting therapy success. Combination therapies are therefore urgently needed. Here, we evaluate the cellular effect of combining a MEK inhibitor with a genotoxic apoptosis inducer. Strikingly, we observed that an activated MAPK pathway promotes in several melanoma cell lines the pro-apoptotic response to genotoxic stress, and MEK inhibition reduces intrinsic apoptosis. This goes along with MEK inhibitor induced increased RAS and P-AKT levels. The protective effect of the MEK inhibitor depends on PI3K signaling, which prevents the induction of pro-apoptotic PUMA that mediates apoptosis after DNA damage. We could show that the MEK inhibitor dependent feedback loop is enabled by several factors, including EGF receptor and members of the SPRED family. The simultaneous knockdown of SPRED1 and SPRED2 mimicked the effects of MEK inhibitor such as PUMA repression and protection from apoptosis. Our data demonstrate that MEK inhibition of BRAF^V600E^-positive melanoma cells can protect from genotoxic stress, thereby achieving the opposite of the intended anti-tumorigenic effect of the combination of MEK inhibitor with inducers of intrinsic apoptosis.

## INTRODUCTION

Melanoma constitutes a very heterogenous cancer type which stands out by its high plasticity. However, a common feature of most melanomas is the activation of the MAPK pathway, which is found in more than 80% of melanomas. In many cases, this is attributed to the expression of oncogenic BRAF which is located directly upstream of MEK. Consequently, the MAPK pathway emerged as a promising therapeutic target. In patients carrying the oncogenic BRAF variant BRAF^V600E^, treatment with the second generation BRAF inhibitors vemurafenib (PLX4032) and dabrafenib (GSK2118436) have led to significant improvement of progression-free and overall survival and thus constitute the most successful treatment strategies since decades [1-3]. Highly increased target protein specificity as well as a strong potency to inhibit MAPK signaling are responsible for this success [4]. However, despite the striking results that were achieved, emerging inhibitor resistance leads to high relapse rates. Secondary reactivation of the MAPK pathway is a commonly observed resistance phenomenon which is mediated by acquired mutations in ERK1/2 upstream components such as NRAS, MEK or BRAF itself [5-7] or by overexpression of MEK effectors such as COT and several receptor tyrosine kinases [5, 8-10]. Direct inhibition of MEK circumvents this problem, as it directly prevents ERK1/2 activation, and thus the application of MEK1/2 inhibitors such as selumetinib (AZD6244) and trametinib (GSK1120212) is pursued for melanoma treatment as well [11, 12]. Indeed, patients preselected for the BRAF^V600E^ mutation showed an improved response to selumetinib or trametinib when compared to standard chemotherapy [11, 12]. The combination of BRAF and MEK inhibitors constitutes an additional clinical strategy and leads to a better progression free survival than BRAF inhibitor monotherapy. Still, the development of resistant tumors as well as a significant group of non-responders remains a pressing problem [13]. It is generally believed that the strong activation of the MAPK pathway which is observed in most melanomas mediates a strong anti-apoptotic response and limits the efficiency of DNA damaging agents [14]. As a consequence, combinations of MAPK pathway inhibitors and chemotherapeutic agents or other pathway inhibitors arise as rationale and are already tested in clinical trials (http://clinicaltrials.gov).

Here we describe the effect of the combination of a MEK inhibitor and, representative for a genotoxic apoptosis inducer, the DNA damaging agent cisplatin in a panel of melanoma cell lines. Unexpectedly, we found that MEK inhibition leads to the protection from cisplatin-induced apoptosis in some cell lines. This was caused by a MEK-inhibitor induced activation of the PI3K/AKT pathway, resulting in transcriptional repression of the pro-apoptotic gene *BBC3,* encoding PUMA. Mechanistically, MEK inhibition relieved several negative feedback loops which include SPRY and SPRED proteins and resulted in enhanced RAS signaling. Receptor tyrosine kinases were involved in this mechanism.

Our data demonstrate for the first time that MAPK pathway inhibition of BRAF^V600E^-mutated melanoma can not only lack an efficient pro-apoptotic effect, but even allows a better survival in presence of a classical inducer of intrinsic apoptosis. As a consequence, MAPK pathway inhibition can even worsen the outcome of melanoma treatment under certain conditions.

## RESULTS

### MEK inhibition can protect melanoma cells from genotoxic apoptosis

**Figure 1:**
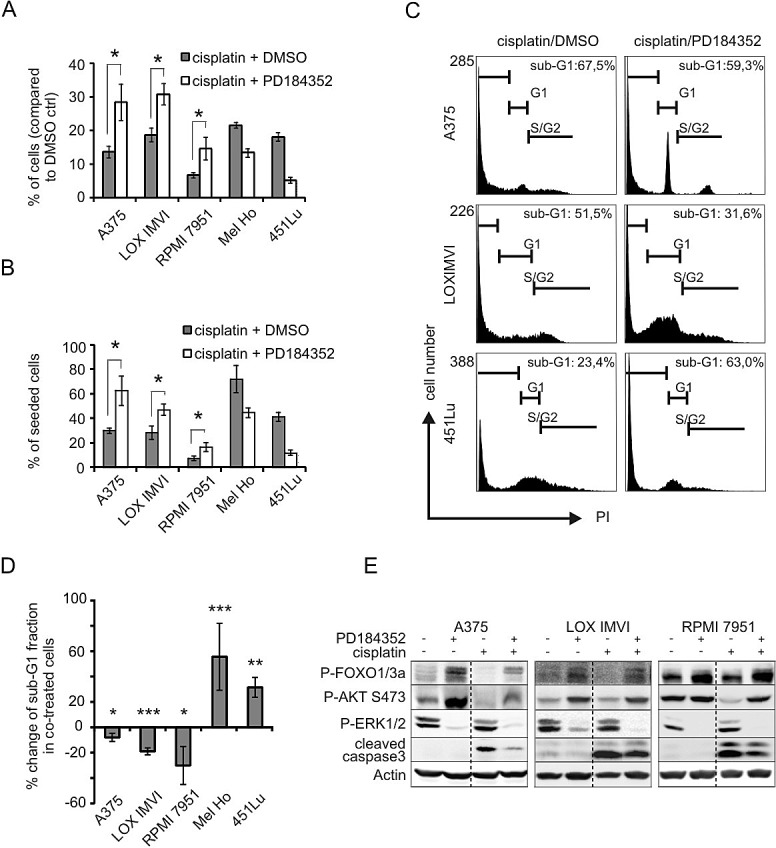
MEK inhibition can protect from cisplatin-induced apoptosis **A**: Surviving cells after cisplatin treatment in presence of DMSO or PD. An equal number of cells was seeded, and cells were treated with cisplatin (10 μM), PD (2 μM) or DMSO as indicated for 48h. The number of living cells was determined at the end of the experiment, and the graph depicts the percentage of cell number compared to the DMSO treated control in absence of cisplatin. Data were derived from two experiments each performed in triplicates. **B**: as **A**, but data are presented in % of seeded cells in order to evaluate the reduction of the original cell number. **C**: FACS profile of A375, LOX IMVI and 451Lu cells cultivated with cisplatin (10 μM) in presence of DMSO or PD (2 μM) for 48h. **D**: Percent change of sub-G1 fraction of cells co-treated for 48h with PD (2 μM) and cisplatin (10 μM), compared to cisplatin alone. Data are derived from two experiments each performed in triplicates. **E**: Indicated cell lines were treated with cisplatin (10 μM) and PD (2 μM) or DMSO for 48 hours. Cell lysates were blotted and levels of P-FOXO1/3a (Thr24/32), P-AKT (Ser473), P-ERK1/2 (Thr202/Tyr204), cleaved caspase 3, and β-actin were determined.

Most melanoma cell lines are susceptible to inhibition of BRAF or MEK. Accordingly, MEK inhibition led to apoptosis and growth reduction in all cell lines from our melanoma cell panel (Figure S1A-C). However, intrinsic or acquired resistance is a major problem in the clinic, thus providing a reason to combine MEK inhibitors with other anticancer drugs such as chemotherapeutic agents. We therefore investigated whether the anti-tumorigenic effect of MEK inhibition could be enhanced by combination with an apoptosis inducer. Chemotherapeutic agents including platinum compounds are applied in combination therapies in clinical trials for cutaneous and uveal melanomas (www.clinicaltrials.gov). As cisplatin is a well-described DNA damaging compound which activates the intrinsic apoptosis pathway, we used it as representative genotoxic apoptosis inducer. We tested the effect of combining the non-competitive MEK inhibitor PD184352 (in short: PD) with cisplatin in five BRAF^V600E^-mutated melanoma cell lines. PD inhibits MAPK activity with IC_50_ values ranging from 100 to 500 nM [15], and we chose a concentration of 2 μM of the inhibitor to efficiently block MAPK signaling (Figure S1A). In all cell lines, cisplatin alone led to a strong reduction of cell number after two days of treatment compared to the DMSO control which was allowed to grow in absence of cisplatin (Figure 1A, gray bars). However, three cell lines showed unexpectedly an enhanced cell number when they were treated with PD in addition to cisplatin (Figure 1A, white bars). To estimate the degree of cisplatin induced cell death, we related the counted cell numbers to the number of seeded cells before treatment (Figure 1B). A decreased rate of cisplatin induced cell death was responsible for the relative increase in cell number in the PD treated melanoma cells A375, LOX IMVI and RPMI 7951 (Figure 1C-E). All three cell lines show only weak apoptosis induction by PD alone (Figure S1C). In Mel Ho and 451Lu cells, which display high apoptosis induction by PD alone (Figure S1C), the combination of PD and cisplatin had an additive inhibitory effect (Figure 1B-D).

As cisplatin is a known inducer of the MAPK pathway [16], thus possibly affecting the efficacy of PD, we determined the extent of ERK1/2 activation after PD treatment in absence or presence of cisplatin. An efficient inhibition of ERK1/2 phosphorylation was detected under all conditions (Figure 1E). Interestingly, the PD-mediated apoptosis-protective effect observed in A375, LOX IMVI, and RPMI 7951 cells went along with a solid increase in P-AKT levels in both absence and presence of cisplatin, indicating AKT activation (Figure 1E). Activation of AKT was furthermore supported by the observation that phosphorylation of the transcription factors FOXO1/3a were enhanced at the AKT-dependent positions Thr24 and Thr32. None of these effects were detected in the cell lines 451Lu and Mel Ho which are highly sensitive to MEK inhibitor (Figure S3). Importantly, the structurally unrelated MEK inhibitor U0126 had a similar effect as PD184352 on cisplatin sensitivity and AKT phosphorylation, as exemplified for A375 cells (Figure S2A,B).

### MEK inhibition by PD can activate AKT in a PI3K dependent manner

**Figure 2:**
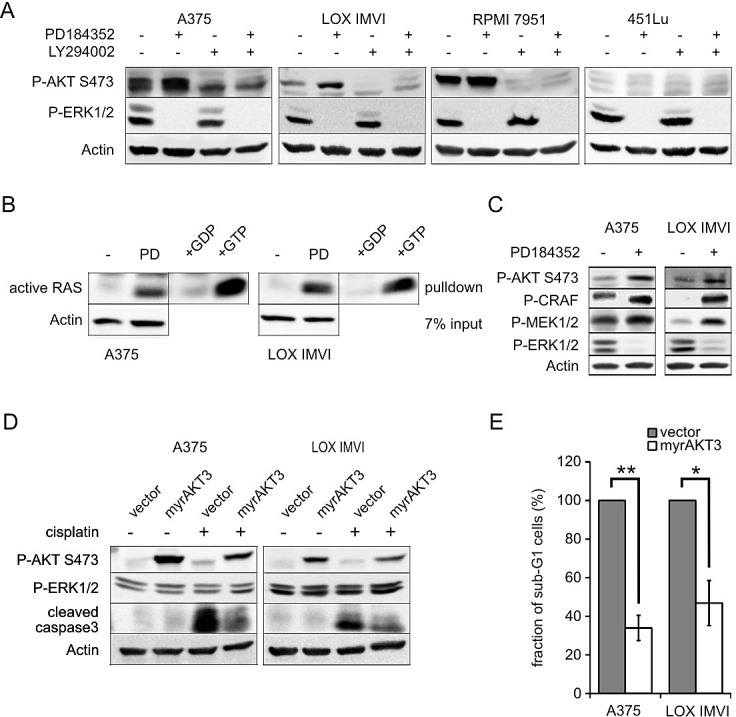
Activated AKT pathway mediates the protective effect of MEK inhibition **A**: A375, LOX IMVI, RPMI 7951 and 451Lu cells were treated with PD (2 μM) and LY294002 (10 μM) as indicated for 24 hours. Cell lysates were blotted and analyzed for presence of P-AKT (Ser473), P-ERK1/2 (Thr202/Tyr204), and β-actin. **B**: A375 and LOX IMVI cells were treated with PD (2 μM) for 24 hours. Active RAS was precipitated from whole cell lysates, and RAS levels were determined. Lysates treated with GDP or GTP served as negative and positive control, respectively. Input lysates were blotted and analyzed for presence of β-actin. **C**: Cells were treated as described in B, and lysates were analyzed by western blot. Levels of P-AKT (Ser473), P-CRAF (Ser259), P-MEK1/2 (Ser217/221) and P-ERK1/2 (Thr202/Tyr204) were determined. β-actin served as loading control. **D**: A375 and LOX IMVI cells were infected with myrAKT3 or vector control and were treated with cisplatin (10 μM) for 2 days. Cell lysates were blotted and analyzed for presence of P-AKT (Ser473), P-ERK1/2 (Thr202/Tyr204), cleaved caspase 3 and β-actin. **E**: Cells and supernatant of myrAKT3 or vector control expressing cells were collected after 2 days of cisplatin (10 μM) treatment, DNA content was stained using propidium iodide and cell cycle profiles were determined. The figure shows the relative rate of cell death. The proportion of cells in sub-G1 of empty vector-transfected cells served as control and were set as 100%.

To check whether Ser473 phosphorylation after MEK inhibition is a consequence of PI3K activation, we applied the PI3K inhibitor LY294002 in addition to PD. Elevated P-AKT Ser473 levels could be prevented by additional administration of the PI3K inhibitor in A375, LOX IMVI and RPMI 7951 cells (Figure 2A). In 451Lu cells, the low P-AKT levels did not change after either PD or LY294002 treatment. These data indicate that MEK inhibitor induced AKT activation is mediated in a PI3K dependent manner. To determine if PI3K activation goes along with increased activity of its upstream activator RAS, we performed a RAS GTPase assay (Figure 2B). While active, GTP-bound RAS was virtually undetectable in DMSO-treated A375 and LOX IMVI cells, MEK inhibition led to robust RAS activation. The extent of this activation correlated with the levels of activated AKT and CRAF, which are induced downstream of RAS (Figure 2C). In addition, MEK1/2 phosphorylation at positions Ser117/221 was elevated (Figure 2C). It is important to note that PD184352 prevents MEK1/2 activation, but still allows phosphorylation at Ser117/221 [6, 17], which might thus accumulate when upstream signaling is enhanced.

### The PI3K/AKT pathway mediates the protective effect of MEK inhibitor treatment

To address whether the observed AKT activation could be the reason for conveying the anti-apoptotic effect, we overexpressed constitutively active myristoylated AKT3 (myrAKT3) in melanoma cell lines. Activated AKT could be visualized by an increase in P-AKT (Ser473) levels (Figure 2D). P-ERK1/2 levels were not affected. In presence of cisplatin, apoptosis was reduced in myrAKT3-expressing cells, as demonstrated by reduced levels of cleaved caspase 3 (Figure 2D) and a strong decrease of the sub-G1 fraction compared to control cells (Figure 2E). Altogether, similar to MEK inhibition, overexpression of myrAKT3 enhanced the resistance to genotoxic stress. Conversely, pharmacological inhibition of PI3K by GDC-0941 enhanced the inhibitory effect of cisplatin both in absence and in presence of MEK inhibitor (Figure S4B). In absence of cisplatin, GDC-0941 also inhibited cell growth in both cell lines, and this effect was further enhanced by PD in A375 cells (Figure S4A).

### Reduction of PUMA mediates the protective effect of PD towards cisplatin

**Figure 3:**
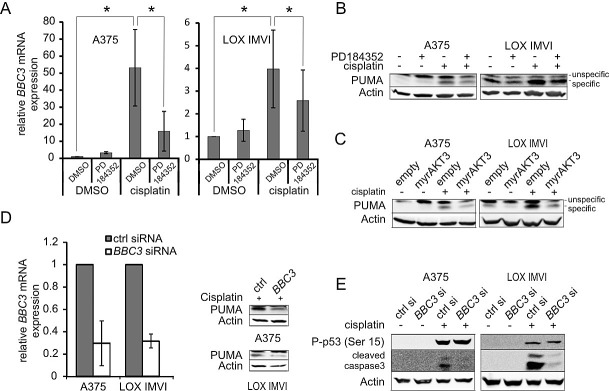
PUMA contributes to cisplatin induced apoptosis and is negatively regulated by PD-induced AKT **A**: A375 and LOX IMVI cells were treated with PD (2 μM) or the solvent DMSO for 24h in absence or presence of cisplatin (10 μM). Levels of *BBC3* mRNA were determined by real-time PCR. Error bars: SD of three independent experiments, each done in triplicate. **B**: As A, but PUMA protein levels were determined by western blot analysis. β-actin served as loading control. **C**: Determination of PUMA protein levels in A375 and LOX IMVI transfected with a control vector or the myrAKT3 overexpression vector. Where indicated, cells were treated with cisplatin (10 μM) for 24h. β-actin served as loading control. **D**: A375 and LOX IMVI cells were treated with *BBC3* siRNA. 24h after transfection, cells were treated with cisplatin (10 μM) for 24h. Knockdown was confirmed by real-time analysis (left) and protein blot (right), as described above. **E**: Western blot showing the induction of P-p53 (Ser15) and cleaved caspase 3 in control and *BBC3* knockdown cells in absence or presence of cisplatin (10 μM, 24h).

To find out how MEK inhibition influences genotoxic apoptosis, we screened a human apoptosis primer library and found a strong cisplatin-mediated induction of *BBC3* (encoding PUMA), which was reduced by PD (Figure 3A, B) as well as U0126 (Figure S2C). The *BBC3* gene product PUMA is a BH3-only, pro-apoptotic member of the BCL2 family. PUMA is involved in the induction of apoptosis after DNA damage [18] and PUMA levels are strongly reduced in human melanomas, indicating the importance of a tight control of PUMA levels during melanomagenesis [19]. Similar to MEK inhibition, the expression of myrAKT3 reduced PUMA levels in presence of cisplatin (Figure 3C). To further examine the role of PUMA in cisplatin-induced apoptosis, we performed siRNA-mediated knockdown of *BBC3*. The knockdown diminished the cisplatin dependent induction of *BBC3* mRNA** and PUMA protein levels to a similar extent as the MEK inhibitor (compare Figure 3D and 3B). In both tested cell lines, PUMA depletion reduced the levels of cleaved caspase 3 without affecting DNA damage, as judged by unchanged levels of the DNA damage indicator P-p53 (Ser15) (Figure 3E).

### Receptor tyrosine kinases mediate MEK inhibitor-induced AKT activation

Receptor tyrosine kinases (RTK) are efficient inducers of RAS, CRAF and AKT, which are all induced in response to the observed MEK inhibition. RTKs have been implicated in drug resistance in melanoma and are involved in mediating the increased invasion phenotype which is seen in some melanoma cells after MEK inhibition [20]. Thus, we tested whether they are also involved in our apoptosis-regulating crosstalk mechanism. We first checked whether the ERK1/2-PI3K crosstalk occurs under conditions in which ERK1/2 can be specifically activated by receptor tyrosine kinase signaling. To accomplish this, we used a murine melanocyte cell line which is transgenic for an engineered chimeric EGFR construct (Figure S5A). When these cells are starved by cultivation under reduced serum conditions, they become quiescent, but can be specifically stimulated by EGFR activation, resulting in proliferation and migration [21-23]. EGF treatment led to strong activation of ERK1/2 and AKT in the melanocytes (Figure S5A). In presence of MEK inhibitor, AKT signaling was enhanced and was maintained for a much longer timespan compared to the controls. These data indicate that RTK signaling can mediate the ERK1/2-PI3K crosstalk, implying a possible involvement of RTKs in PD-mediated AKT activation in the melanoma cell lines. A comparable experiment was therefore conducted with the human melanoma cell lines A375 and LOX IMVI. As these cells express many different RTKs which could potentially be involved, they were first starved and then treated with 10% FCS. FCS contains numerous growth factors [24], and by using FCS we intended to reach simultaneous activation of several different receptors (Figure S5B). Under starved conditions, phosphorylated AKT was almost invisible in both cell lines. In contrast, there was a robust P-ERK1/2 signal, which is attributed to the activating BRAF^V600E^ mutation. FCS stimulation led to enhanced AKT in both cell lines, and, in case of A375 cells, to reduced ERK1/2 signaling over time. Again, MEK inhibition resulted in enhanced and longer lasting AKT activation in both melanoma cell lines. These data indicate that the MEK inhibitor dependent PI3K pathway activation can be mediated by growth stimuli which are present in FCS. In case of A375 cells, MEK inhibition even induced AKT phosphorylation in absence of FCS (Figure S5B, lane 2).

**Figure 4:**
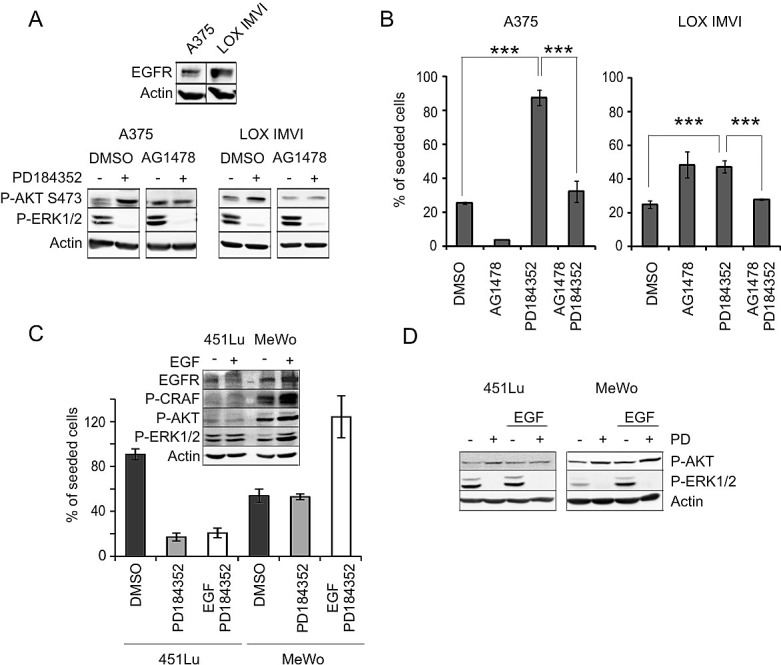
EGFR signaling is implicated in MEK inhibitor induced AKT activation **A**: Inhibition of EGFR abrogates PD induced AKT phosphorylation. Upper image: Western blot showing expression of EGFR in A375 and LOX IMVI cells. Lower image: Protein blot of A375 and LOX IMVI cells treated for 24h with DMSO, PD (2 μM), AG1478 (20 μM) or both inhibitors in combination. Levels of P-ERK1/2 (Thr202/Tyr204) and P-AKT (Ser473) were determined. β-actin served as reference. **B**: Quantification of cisplatin-treated A375 and LOX IMVI cells in presence of MEK- and EGFR inhibitors. 1×10^5^ cells were seeded and treated with PD (2 μM), AG1478 (10 μM), or a combination of both in presence of cisplatin (10 μM). The number of surviving cells was determined 48h later. Data are presented as % of seeded cells and are derived from two experiments each performed in triplicates. **C** (upper image): Effect of EGF stimulation on the c-RAF/ERK1/2 and PI3K pathways in 451Lu and MeWo cells. Cells were stimulated with EGF for 15 minutes, and levels of EGFR, P-CRAF (Ser259), P-AKT (Ser473) and P-ERK1/2(Thr202/Tyr204) were determined by western blot analysis. β-actin served as loading control. C (lower image): Effect of EGF stimulation on sensitivity of 451Lu and MeWo cells towards cisplatin in presence of PD. 1×10^5^ cells were seeded and treated with DMSO, PD (2 μM), or PD (2 μM) in combination with 8 nM EGF in presence of cisplatin (10 μM). The number of surviving cells was determined 48h later. Data are presented as % of seeded cells and are derived from triplicates. **D**: Western blot showing the effect of PD treatment (2 μM) on P-AKT and P-ERK1/2 in control and EGF-treated 451Lu and MeWo cells. Cell lysates were blotted and analyzed for presence of P-AKT (Ser473), P-ERK1/2 (Thr202/Tyr204), and β-actin.

It was previously reported that transcriptional induction of IGF1 can occur in response to BRAF inhibition [9]. To check whether MEK inhibition could also affect expression of RTK ligands, we investigated the RNA levels of EGF, IGF1, PDGF and HGF in A375 and LOX IMVI cells. HGF was detected in none of the two cell lines (data not shown), but EGF, IGF1 and PDGF were expressed under standard growth conditions. While MEK inhibition had no effect on any of the investigated ligands in LOX IMVI cells, EGF and IGF1 were significantly induced by PD in A375 cells (Figure S5C). In case of EGF, RNA levels were increased 9-fold. EGFR is expressed at low or moderate levels in many melanoma cells [25] and was also detected in A375 and LOX IMVI cells (Figure 4A). The inhibition of EGFR with the small molecule inhibitor AG1478 had different effects on the cell lines: A375 cells were sensitized to cisplatin, whereas LOX IMVI cells were protected from cisplatin-induced apoptosis to a similar extent as in presence of PD (Figure 4B). However, EGFR inhibition resensitized both cell lines to cisplatin in presence of PD and abolished PD-mediated AKT activation (Figure 4A, B).

We also investigated the impact of EGFR activation on two cell lines which show very low (MeWo) or high (451Lu) levels of apoptosis induction in response to PD treatment (see Figure S1B, C). Both cell lines express low levels of EGFR, but in MeWo cells they were slightly higher compared to 451Lu (Figure 4C). Interestingly, EGF stimulation led to an increase in downstream RAF/ERK1/2 and PI3K/AKT pathways in MeWo, but not in 451Lu cells (Figure 4C and, from an independent experiment, Figure S5D) Furthermore, EGF enhanced survival in presence of cisplatin and PD only in MeWo cells (Figure 4C). Consequently, EGF stimulation might be involved in PD-dependent AKT activation and protection from cisplatin in MeWo cells (Figure 4C, D).

### MEK inhibition relieves several feedback loops which contribute to PI3K pathway activation and apoptosis resistance

**Figure 5:**
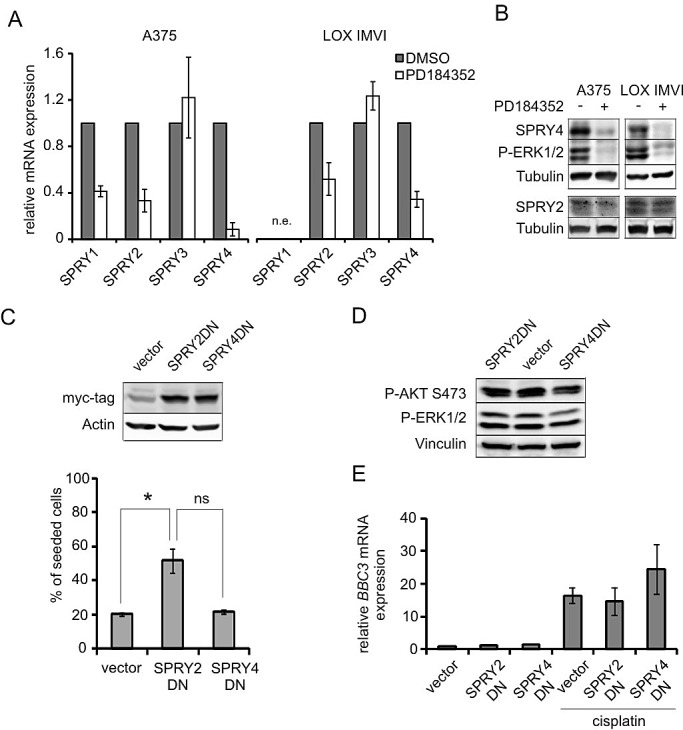
Downregulation of functional *SPRY2* contributes to apoptosis resistance in a PUMA-independent manner **A**: Real-time PCRs displaying the expression of *SPRY1-4* mRNAs in A375 and LOX IMVI cells after MEK inhibition. Cells were treated with PD (2 μM) or DMSO for 24h. Relative levels of *SPRY1-4* mRNAs were normalized to *RS14* levels and DMSO-treated cells were set as reference. Error bars: SD of three independent experiments. n.e.: not expressed. **B**: Western blot displaying SPRY2 and SPRY4 levels in response to MEK inhibition as described in A. Tubulin served as loading control. C, upper image: Western blot showing the expression of myc-tagged *SPRY2DN* and *SPRY4DN* in A375 cells. β-actin served as loading control. **C**, lower image: A375 cells expressing SPRY2DN, SPRY4DN or empty control vector were seeded at equal density and were treated with cisplatin. The number of living cells was determined 48h after treatment. Data are presented as % of seeded cells and are derived from two experiments each performed in triplicates. **D**: Western blot displaying P-AKT (Ser473) and P-ERK1/2 (Thr202/Tyr204) levels in A375 control, SPRY2DN or SPRY4DN expressing cells. Vinculin served as loading control **E**: *BBC3* (PUMA) mRNA levels in A375 control, SPRY2DN or SPRY4DN expressing cells in absence or presence of cisplatin (10 μM, 24h). Data are derived from two independent experiments, each done in triplicates.

**Figure 6:**
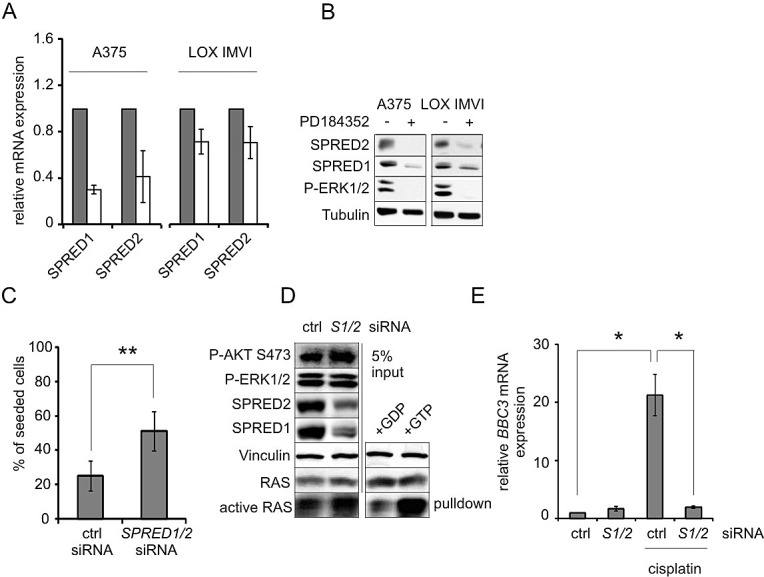
Downregulation of SPRED1/2 mimicks the effect of MEK inhibitor on PUMA and apoptosis resistance **A**: Real-time PCRs displaying the expression of *SPRED1-2* mRNAs in A375 and LOX IMVI cells after MEK inhibition. Cells were treated with PD (2 μM) or DMSO for 24h. Relative levels of *SPRED mRNAs* were normalized to *RS14* levels and DMSO-treated cells were set as reference. Error bars: SD of two independent experiments, each done in triplicate. **B**: Western blot displaying SPRED1 and SPRED2 levels in response to MEK inhibition as described in A. Tubulin served as loading control. **C**: A375 cells were transfected with control siRNA or siRNAs directed against *SPRED1* and *SPRED2*. After 24h, cells were treated with 10 μM cisplatin for 48h, and cells were counted. Data are presented as % of seeded cells and are derived from two experiments each performed in triplicates. **D**: A375 cells were transfected with control siRNA or siRNAs directed against *SPRED1* and *SPRED2*. Active RAS was precipitated from whole cell lysates, and RAS levels were determined. Lysates treated with GDP or GTP served as negative and positive control, respectively. Input lysates were blotted and analyzed for presence of P-AKT (Ser473), P-ERK1/2 (Thr202/Tyr204), SPRED1, SPRED2, and vinculin as loading control. **E**: *BBC3* (PUMA) mRNA levels in A375 cells transfected with control (ctrl) or *SPRED1/2* (*S1/2*) siRNA in absence or presence of cisplatin (10 μM, 24h). Data are derived from three independent experiments, each done in triplicates.

The MAPK pathway is prone to tight regulation mechanisms which guarantee the transient nature of pathway activation under physiological conditions. Dual specific phosphatases (DUSP), SPRY and SPRED family proteins are induced by the RAF/MEK/ERK1/2 pathway [22, 26]. SPRY and SPRED proteins can exert their function downstream of RTKs and were reported to be involved in RAS regulation [27, 28]. Thus, we hypothesized that SPRY or SPRED proteins are involved in MEK inhibitor dependent protection from apoptosis. Along these lines, MEK inhibition reduced RNA and protein levels of *SPRY2*, *SPRY4* (Figure 5A, B) as well as *SPRED1* and *SPRED2* (Figure 6A, B) in A375 and LOX IMVI cells. In addition, *SPRY1* was reduced on RNA level in A375 cells (Figure 5A). It was previously demonstrated that a reduction of SPRY genes increases RAS downstream signaling in melanoma [29]. To investigate the impact of SPRY2 and -4 on protection from apoptosis, we overexpressed dominant negative constructs of both proteins in A375 cells. The mutants destroying Tyr55 or Tyr53 of SPRY2 and SPRY4, respectively, are incapable of mediating RTK feedback inhibition, but instead act as dominant negative protein versions [30-32]. SPRY2DN, but not SPRY4DN, enhanced melanoma cell survival in presence of cisplatin (Figure 5C). However, the levels of activated AKT or cisplatin-induced *BBC3* were neither affected by SPRY2DN nor by SPRY4DN (Figure 5D, E), suggesting that the protection from apoptosis by SPRY2DN occurs via an AKT/PUMA-independent mechanism.

In addition, SPRED1 and -2 are established negative regulators of the RAS/RAF/ERK1/2 pathway. To test whether SPRED1 and -2 are involved in mediating the observed ERK1/2/AKT crosstalk, we knocked down both genes simultaneously. Again, we observed a protection from genotoxic apoptosis (Figure 6C, Figure S6). This went along with a modest enhancement of active RAS and AKT (Figure 6D) and with the prevention of cisplatin-induced *BBC3* (Figure 6E).

**Figure 7:**
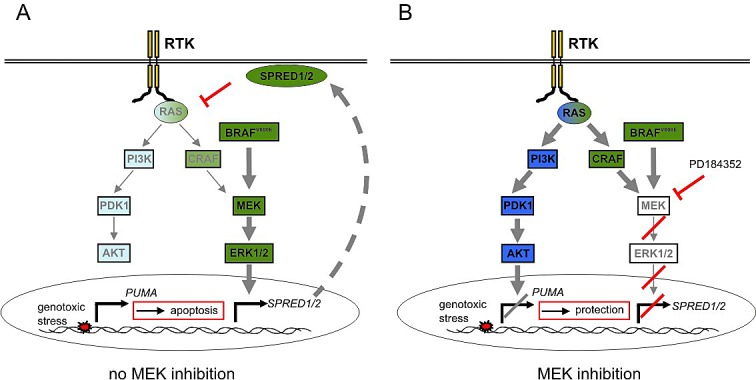
Scheme of the MEK inhibitor induced crosstalk mechanism **A**: In absence of MEK inhibitor, BRAF^V600E^ activation leads to the ERK1/2-dependent induction of SPRED genes, which block RAS signaling downstream of RTKs in melanoma cells. This results in low PI3K pathway activity. When cells are exposed to genotoxic stress, PUMA is induced and cells undergo apoptosis. **B**: Application of MEK inhibitor abolishes the expression of *SPRED1* and *SPRED2* and thereby relieves the negative feedback on RTK signaling. As a result, the PI3K pathway is activated. This prevents the induction of the apoptosis promoter PUMA in response to genotoxic stress and allows enhanced survival and protection from apoptosis.

In summary, our data reveal a complex RTK-associated and SPRED1/2-mediated feedback network which can be relieved by MEK inhibition and thereby protects from DNA damage induced apoptosis in melanoma cells (summarized in Figure 7).

## DISCUSSION

In the present study we show that inhibition of the MAPK pathway has the potential to protect melanoma cells from apoptosis induced by genotoxic damage. This effect was conveyed by activation of the PI3K pathway which occurred shortly after the start of MEK inhibition and was mediated by multiple factors. Several negative feedback components including SPRY and SPRED proteins were affected by MEK inhibition. SPRED1 is a very efficient regulator of RAS and certain inherited SPRED1 mutants cause the Legius syndrome which belongs to the so-called “rasopathies” – diseases characterized by enhanced RAS signaling [33]. SPRED1 exerts its function by binding and inhibiting neurofibromin 1 (NF1), one of the major RAS GTPase activating proteins [28]. NF-1 was recently identified as top hit in an shRNA screen designed to identify genes whose disruption convey BRAF inhibitor resistance in melanomas [34]. It is mutated in a significant number of melanomas, including those with BRAF inhibitor resistance [35, 36]. We could demonstrate that siRNA-mediated reduction of functional SPRED1 and 2 siRNA aids in enhancing RAS activity and reducing PUMA-dependent apoptosis by genotoxic stress. Furthermore, reduction of SPRY2 also contributed to protection from apoptosis, as dominant negative SPRY2 doubled the fraction of cells which survived cisplatin treatment. Interestingly, this protective effect did not go along with AKT induction or PUMA regulation, suggesting an independent additional mechanism which might contribute to the observed MEK inhibitor dependent effect. Of note, it was recently reported that a reduction of SPRY2 by either BRAF inhibition or siRNA enhanced the responsiveness to exogenous ligands and thereby enhanced mitogenic signaling [29], thus indicating that a reduction of SPRY2 can provide conditions which also facilitate resistance to BRAF inhibitor monotherapy.

However, we also observed the downregulation of SPRY2 and 4 as well as SPRED1 and -2 in a melanoma cell line (451Lu) which is MEK inhibitor sensitive and does not display enhanced AKT activation by PD (Figure S7). We propose that the repression of SPRY and SPRED genes is a common phenomenon in MEK inhibited melanoma cells, but that it can only contribute to drug resistance and protection from genotoxic damage in presence of a permissive cellular context. Melanoma cells which express a certain set of receptor tyrosine kinases supposedly provide this permissive condition. Our data indicate that EGFR belongs to the potent RTKs which permit the MEK-inhibitor dependent AKT activation, followed by enhanced apoptosis resistance.

In BRAF^V600E^ mutant colorectal cancer cells, which generally express higher levels of EGFR than melanomas, activation of EGFR and downstream pathways occurs in response to vemurafenib and is responsible for resistance towards this BRAF inhibitor [37]. EGFR-expressing cells seem to be particularly prone to feedback activation in several cancer cell types, such as melanoma (our data and [38]), colon cancer [37] and breast cancer [39]. Recent studies show that EGFR expression occurs in a significant percentage of BRAF resistant melanomas, and receptor activation, though disadvantageous under normal conditions, becomes beneficial when the MAPK pathway is blocked in BRAF^V600E^ positive melanomas [40]. Also, FOXD3-dependent upregulation of the EGFR family member ERBB3 was described after BRAF- or MEK inhibition in melanoma cells, resulting in enhanced responsiveness to ERBB3 ligands and the sensitization towards the BRAF inhibitor PLX4720 by lapatinib [41]. Therefore, it is highly likely that ERBB3 also belongs to the factors which determine the permissive environment.

Apart from receptor expression, the corresponding set of ligands has to be expressed and shedded in order to allow the protective effect of MEK inhibition towards genotoxic stress. We found that in MeWo cells only the addition of EGF caused the protective effect of PD towards cisplatin, indicating that these cells do not produce sufficient ligands to reach autocrine stimulation. In order to produce and secrete sufficient ligands, cells have to express a certain molecular equipment. For example, EGFR ligands can be induced by EGFR itself [42], by RAS [43], or IL-1β [44]. The shedding of EGFR ligands is mainly dependent on the metalloproteases ADAM-17 and ADAM10 [45], the latter being induced by PAX2 in melanoma [46]. Interestingly, Vultur et al. described recently the activation of several RTKs and the downstream signal transducer STAT3 in 20% of MEK-inhibitor treated melanoma cells [20]. They showed that MEK inhibitor induced STAT3 activation results in increased melanoma cell invasion. It is well possible that their reported STAT3-dependent invasion and our AKT-dependent protection from apoptosis are the consequences from similar upstream events.

Mechanistically, we found that AKT-dependent reduction of the pro-apoptotic effector PUMA was involved in the protection from apoptosis. PUMA is a FOXO1/3a target which is strongly induced in presence of genotoxic stress [47], [48] and is implicated in the induction of apoptosis in melanoma [49]. Importantly, PUMA expression correlates inversely with the melanoma malignancy grade, and weak PUMA expression is associated with poorer overall survival [19]. By reducing PUMA levels, MEK-inhibition can therefore evoke unexpected pro-tumorigenic effects, even in BRAF^V600E^-mutant melanomas. It is however possible that apart from PUMA, other AKT downstream targets also contribute to the observed anti-apoptotic effect.

Compensatory PI3K pathway activation might be a serious problem in the clinical setting, where BRAF- and MEK inhibitors can encounter intrinsic [50-52] as well as acquired [5, 9] PI3K pathway reactivation which is likely involved in tumor relapse. After the tumors reappear, chemotherapeutic and DNA damaging agents are often applied, usually without benefit for the patient. The reason for this might be found, at least partially, in the apoptosis resistance conferred by PI3K pathway activation. Consequently, PI3K pathway inhibitor combination therapies are a promising approach. There are encouraging preclinical data addressing this strategy. In melanoma cell lines, acquired BRAF inhibitor resistance is overcome by simultaneous MEK and PI3K/mTOR inhibition [53]. Along these lines, combined MEK and PI3K/mTORC inhibition also leads to tumor regression in murine melanoma models driven by mutant RAS [54]. In cancer patients, dual inhibition of BRAF/MEK/ERK1/2 and PI3K/AKT/mTOR pathways is in phase I clinical trials [55]. So far, only few melanoma patients were enrolled in this study, and it is too early to state whether the dual inhibition strategy is more successful than BRAF inhibitor monotherapy.

In summary, our data demonstrate that MEK inhibition of BRAF^V600E^-positive melanoma cells can lead to AKT-dependent enhanced apoptosis resistance towards genotoxic stress, implying a careful choice of pathway inhibitors in combination therapies.

## METHODS

### Compounds

The MEK inhibitor PD184352 (in short PD) and GDC-0941 were purchased from Axon Medchem and Selleckchem, respectively. The PI3K inhibitor LY294002, the EGFR inhibitor AG1478 and cisplatin were obtained from Calbiochem. ON-TARGET plus SMART pool siRNA for *BBC3 (PUMA), SPRED1*, *SPRED2* and non-targeting siRNA were from Thermo Scientific. For transfection of siRNA, X-tremeGENE siRNA Transfection Reagent (Roche) was used according to the manufacturer's instructions.

### Cell lines and plasmids

A375, Mel Ho, and SK MEL28 were purchased from ATCC. LOX IMVI, RPMI 7951, UACC-62, M19MEL, SK MEL2, SK MEL5 are part of the NCI-60 panel and were obtained from the NCI/NIH (DCTD Tumor Repository, National Cancer Institute at Frederick, Frederick, Maryland). 451Lu and MeWo cells were kindly provided by Meenhard Herlyn (Wistar Institute, Philadelphia, PA) and Anja Bosserhoff, (Institute of Pathology, University of Regensburg, Germany), respectively. All cell lines were maintained in DMEM with 10% FCS at 37° and 5% CO_2_. HERmrk cells were cultivated as described earlier [56]. Cell lines were authenticated using the PowerPlex 16 DNA typing system (Promega).

The plasmid containing the constitutively active myristoylated AKT3 (pBabe-puroL-Myr-HA-Akt3) was purchased from Addgene (Addgene plasmid 9019). Dominant negative constructs of SPRY2 and SPRY4 (pcDNA3 SPRY2DN, pcDNA3 SPRY4DN) [32] were kindly supplied by Akihiko Yoshimura (original names: pcDNA3 SPRY2 Y53A and pcDNA SPRY4 Y55A) (Kurume, Japan). Truncated dominant negative versions of SPRED1 and SPRED2 (SPRED1DN, SPRED2DN) were designed as described earlier [57] and cloned in p201iEP for lentiviral delivery.

### Protein extraction and Western blot

Cells were lysed in lysis buffer (20 mM HEPES (pH 7.8), 500 mM NaCl, 5 mM MgCl_2_, 5 mM KCl, 0.1% deoxycholate, 0.5% Nonidet-P40, 10 mg/ml aprotinin, 10 mg/ml leupeptin, 200 mM Na_3_VO_4_, 1 mM phenylmethanesulphonylfluoride and 100 mM NaF). 30-50 μg of protein lysate was separated by SDS-PAGE and transferred to nitrocellulose membranes. Anti-β-actin (C4) antibody was purchased from Santa Cruz Biotechnology (sc-47778). Antibodies directed against P-ERK1/2 (Thr202/Tyr204), P-AKT (Ser473), P-FOXO1/3a (Thr24/32), P-MEK1/2 (Ser217/221) cleaved caspase 3 (Asp175), P-CRAF (Ser259), PUMA, myc-tag, and P-p53 (Ser15) were purchased from Cell Signaling Technologies. Antibodies against SPRY2 and SPRY4 were purchased from Sigma Aldrich, the SPRED1 antibody was acquired from Thermo Scientific. The antibody against SPRED2 was kindly provided by Kai Schuh (Würzburg, Germany). Generally, the presented protein blots are representative for 2-3 independent experiments.

### RAS activity assay

750 μg of total protein lysate was used to determine the levels of active, GTP-bound RAS using the Active RAS Pull-Down and Detection Kit (Thermo Scientific), according to the manufacturer's instructions.

### RNA isolation and real-time PCR

RNA isolation was performed with peqGOLD Trifast™ solution (PEQlab). Whole RNA (1-4 μg) was reversely transcribed using the RevertAid™ First Strand cDNA Synthesis Kit (Fermentas). Quantitative real-time PCR was performed using a Mastercycler ep realplex™ (Eppendorf). Gene expression was normalized to *RS14*.

### Cell cycle analysis

Cells were harvested and fixed in 70% ETOH at −20°C for at least 24 hours and rinsed twice with PBS. For flow cytometry analysis, cells were treated with 0.5 mg/ml RNase A for 30 min at 37°C before the DNA was stained with 69 mM propidium iodide in 38 mM sodium citrate. Flow cytometry was performed using a Cytomics FC 500 flow cytometer (Beckman Coulter). Results were analyzed using the cxp analysis software (Beckman Coulter).

### Proliferation assay

0.5-1.0 ×10^5^ melanoma cells were seeded in triplicates on six-well dishes. 24 hours after seeding cells were treated with inhibitors and cisplatin as indicated. Media containing the drugs was changed every other day. At indicated time points, all cells were harvested and the number of living cells was determined using a Neubauer hemocytometer at indicated time points, using the trypane blue exclusion method.

### Statistical analyses

Data presented in the bar graphs represent the mean values of at least three independent data points as indicated in the respective figure legends. The error bars represent the standard deviation. Significance was determined using an unpaired two-tailed t-test (*: p≤0.05; **: p≤0.01; ***: p≤0.001).

## SUPPLEMENTARY FIGURES


