# Supplementary material for: The MAPK pathway as an apoptosis enhancer in melanoma
Source: Oncotarget. 2014 Jun 8;5(13):5040–53. doi: 10.18632/oncotarget.2079 (PMC4148120; doi:10.18632/oncotarget.2079)
Supplement: Supplementary file 1 [file oncotarget-05-5040-s001.pdf]

The MAPK pathway as an apoptosis enhancer in melanoma

Supplementary Material

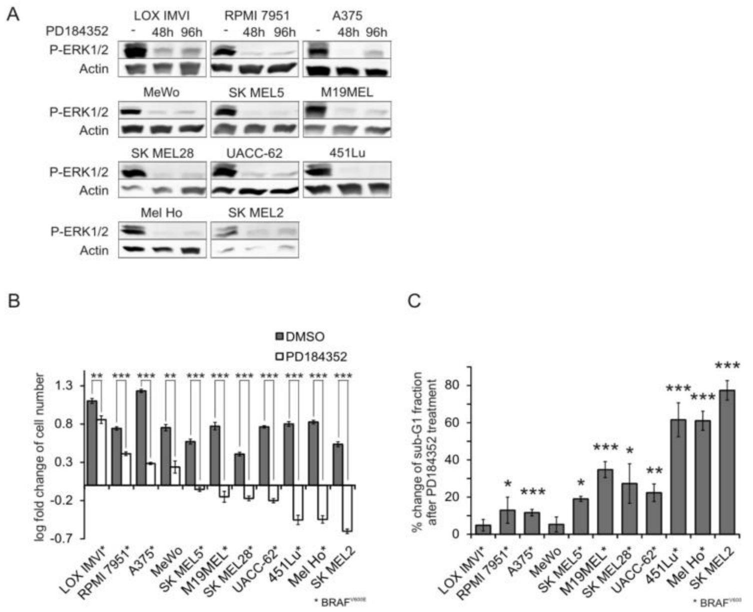

Figure S1: Cellular effect of MEK inhibition on melanoma cells.

A: Protein blot of indicated melanoma cell lines, showing P-ERK1/2 (Thr202/Tyr204) levels after 48h or 96h of treatment with PD (2 µM). β-actin served as internal reference. **B:** Determination of cell growth in response to MEK inhibition. $5x10^4$ cells of each cell line were seeded and treated with PD (2 µM) or the equivalent amount of the solvent DMSO. Number of living cells was determined after four days and blotted as log fold change of cell number (compared to day 0). Error bars: SD of three experiments, each done in triplicates. Please note that growth of the left four cell lines is affected by PD in all cases (e.g. the log fold reduction of LOX IMVI translates into a growth reduction by 40% compared to the control). All values below 0 show a reduction of initial seeded cell number. **C:** Increase of sub-G1 fraction after 96h of PD treatment compared to DMSO. Data represent the mean values of three experiments.

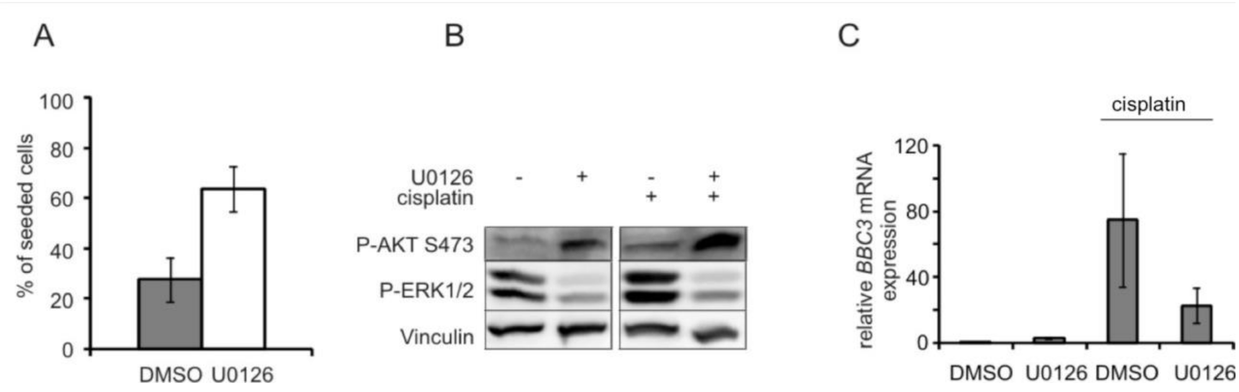

Figure S2: Apoptosis-protective effect by an independent MEK inhibitor

A: A375 cells were seeded at equal density and were treated with DMSO or 5 µM U0126 in absence or presence of cisplatin. The number of living cells was determined 48h after treatment. Data are presented as % of seeded cells and are derived from one experiment performed in triplicates. **B:** A375 cells were treated with cisplatin (10 µM) and U0126 (2 µM) or DMSO for 48 hours. Cell lysates were blotted and levels of P-AKT (Ser473), P-ERK1/2 (Thr202/Tyr204), and vinculin (as loading control) were determined. **C:** *BBC3* (PUMA) mRNA levels in A375 cells treated with DMSO as control or 5 µM U0126 in absence or presence of cisplatin (10 µM, 24h). Data are derived from two independent experiments, each performed in triplicates.

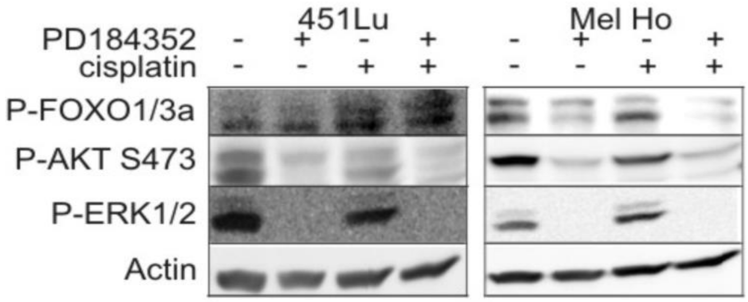

Figure S3: Effect of MEK inhibition and cisplatin on melanoma cells.

451Lu and Mel Ho cells were treated with cisplatin (10 µM) and PD (2 µM) or DMSO for 48 hours. Cell lysates were blotted and levels of P-FOXO1/3a (Thr24/32), P-AKT (Ser473), P-ERK1/2 (Thr202/Tyr204), and β-actin (as loading control) were determined.

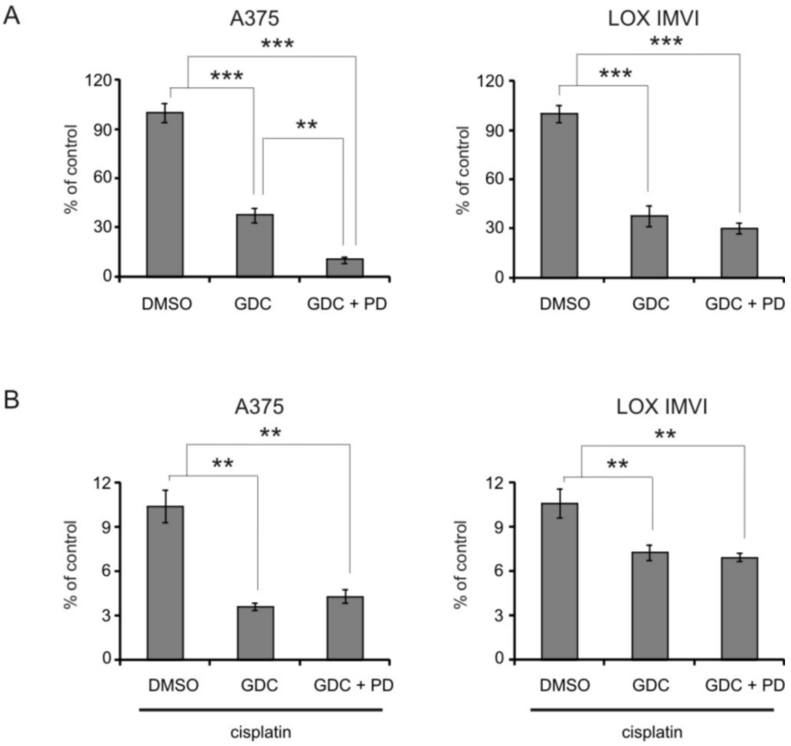

Figure S4: Effect of PI3K inhibition on cisplatin-induced genotoxic stress

A: 5x10^4 cells of A375 and LOX IMVI cells were seeded and treated with DMSO or with the PI3K inhibitor GDC-0941 (4 µM) alone or in combination with MEK inhibitor PD184352 (2 µM), as indicated. Relative amount of living cells was determined after 2 days. **B:** As **A**, but in presence of cisplatin (10 µM). Error bars: SD of two experiments, done in triplicate.

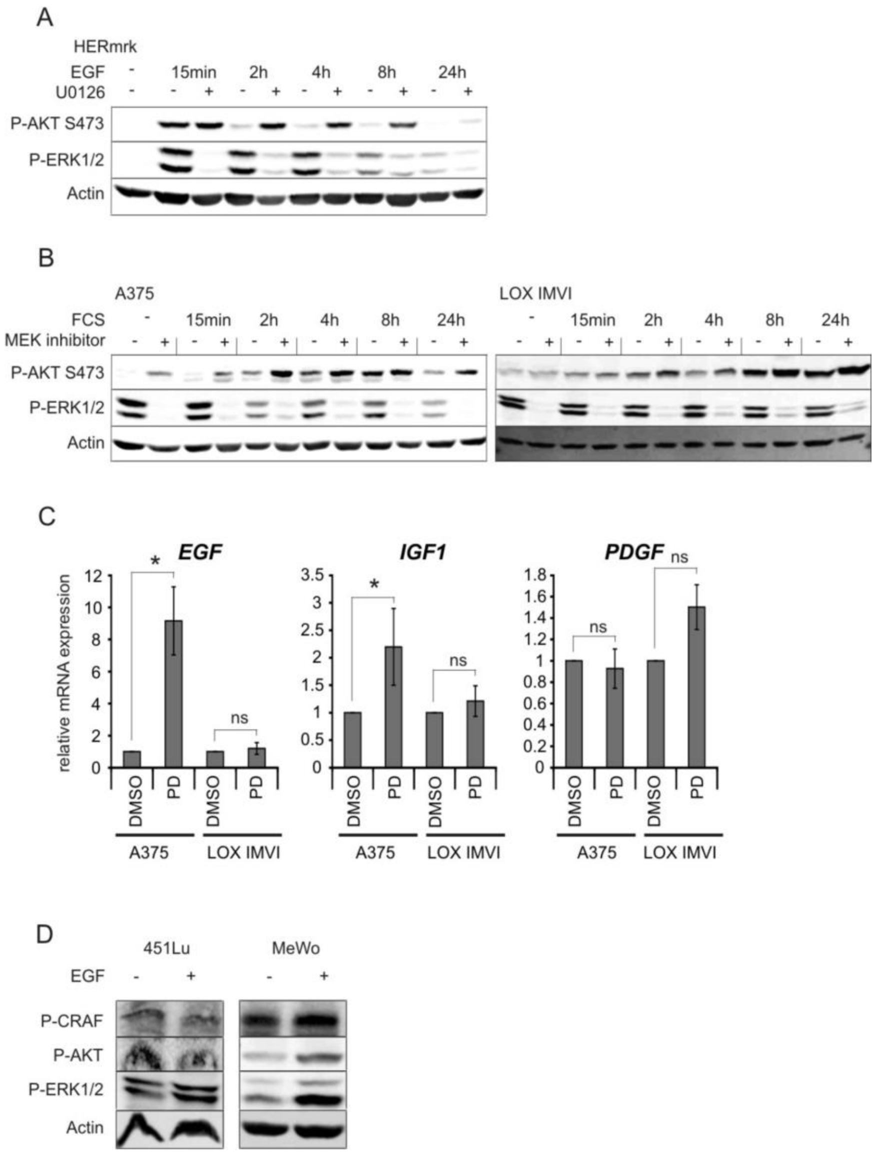

Figure S5: The role of growth factors in PD-mediated AKT activation. A: Kinetics of AKT phosphorylation in response to EGF stimulation. Melan-a melanocytes transgenic for the artificial EGFR construct HERmrk were serum-starved for 24h and treated with EGF (100 ng/ml) in absence or presence of the MEK inhibitor U0126 (10

µM) for the indicated time points. Levels of P-ERK1/2 (Thr202/Tyr204) and P-AKT (Ser473) were determined by western blot. β-actin served as loading control. **B:** Kinetics of AKT phosphorylation in response to FCS stimulation. A375 and LOX IMVI cells were serum-starved for 24h and treated with FCS (10%), in absence or presence of the MEK inhibitors U0126 (10 µM) (A375) or PD (2 µM) (LOX IMVI), for the indicated time points. Levels of P-ERK1/2(Thr202/Tyr204) and P-AKT (Ser473) were determined by western blot. β-actin served as loading control. **C:** Real-time

PCR analysis of *EGF*, *IGF1* and *PDGF* in response to MEK inhibition. A375 and LOX IMVI cells were treated for 24h with DMSO (as control) or with PD (2 µM). The expression changes of PD-treated cells relative to DMSO treated cells were determined. **D:** Effect of EGF stimulation on the c-RAF/ERK1/2 and PI3K pathways in 451Lu and MeWo cells. Cells were stimulated with EGF for 15 minutes, and levels of P-CRAF (Ser259), P-AKT (Ser473) and P-ERK1/2(Thr202/Tyr204) were determined by western blot analysis. β-actin served as loading control.

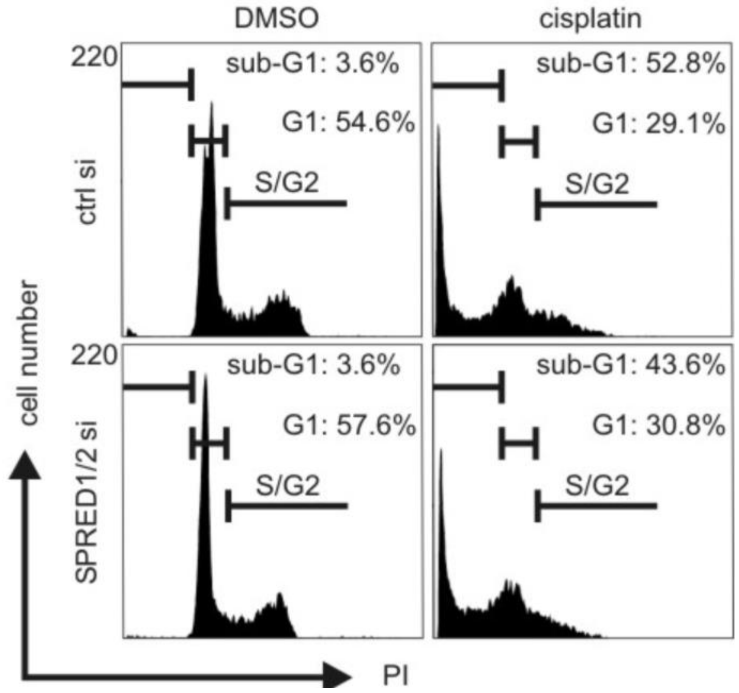

Figure S6: Knockdown of SPRED1/2 weakens the pro-apoptotic effect of cisplatin Determination of cell death in control cells and *SPRED1*- or *SPRED2*-knockdown cells. Cells and supernatant were collected after 48h of cisplatin treatment, DNA content was stained using propidium iodide and cell cycle profiles were analyzed. The experiment was done twice, and the data show representative cell cycle profiles.

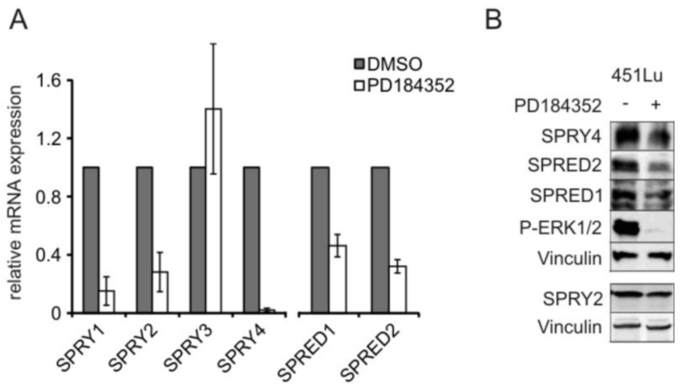

Figure S7: MEK inhibition reduces the expression of SPRY and SPRED proteins in 451Lu cells.

A: Real-time PCRs displaying the expression of *SPRY1-4* (left) and *SPRED1-2* (right) mRNAs in 451Lu cells after MEK inhibition. Cells were treated with PD (2 µM) or DMSO for 24h. Relative levels of *SPRY* and *SPRED mRNAs* were normalized to *RS14* levels and DMSO-treated cells were set as reference. Error bars: SD of two experiments, each done in triplicates. n.e.: not expressed. **B:** Western blot displaying SPRY2, SPRY4, SPRED1 and SPRED2 levels in response to MEK inhibition as described in **A.** Vinculin served as loading control.
